# Histone Deacetylase Inhibitors Sensitize TRAIL-Induced Apoptosis in Colon Cancer Cells

**DOI:** 10.3390/cancers11050645

**Published:** 2019-05-10

**Authors:** Baojie Zhang, Bin Liu, Deng Chen, Rita Setroikromo, Hidde J. Haisma, Wim J. Quax

**Affiliations:** Department of Chemical and Pharmaceutical Biology, Groningen Research Institute of Pharmacy, University of Groningen, Antonius Deusinglaan 1, 9713 AV Groningen, The Netherlands; b.z.zhang@rug.nl (B.Z.); bin.liu@rug.nl (B.L.); d.chen.5@student.rug.nl (D.C.); R.Setroikromo@rug.nl (R.S.); h.j.haisma@rug.nl (H.J.H.)

**Keywords:** HDAC, TRAIL, RGFP966, PCI34051, DLD-1, WiDr, spheroid, death receptor

## Abstract

Tumor necrosis factor-related apoptosis-inducing ligand (TRAIL) is considered as a promising anti-cancer therapeutic. However, many cancers have been found to be or to become inherently resistant to TRAIL. A combination of epigenetic modifiers, such as histone deacetylase inhibitors (HDACi’s), with TRAIL was effective to overcome TRAIL resistance in some cancers. Broad spectrum HDACi’s, however, show considerable toxicity constraining clinical use. Since overexpression of class I histone deacetylase (HDAC) has been found in colon tumors relative to normal mucosa, we have focused on small spectrum HDACi’s. We have now tested agonistic receptor-specific TRAIL variants rhTRAIL 4C7 and DHER in combination with several class I specific HDACi’s on TRAIL-resistant colon cancer cells DLD-1 and WiDr. Our data show that TRAIL-mediated apoptosis is largely improved in WiDr cells by pre-incubation with Entinostat-a HDAC1, 2, and 3 inhibitor- and in DLD-1 cells by RGFP966-a HDAC3-specific inhibitor- or PCI34051-a HDAC8-specific inhibitor. We are the first to report that using RGFP966 or PCI34051 in combination with rhTRAIL 4C7 or DHER represents an effective cancer therapy. The intricate relation of HDACs and TRAIL-induced apoptosis was confirmed in cells by knockdown of *HDAC1*, *2*, or *3* gene expression, which showed more early apoptotic cells upon adding rhTRAIL 4C7 or DHER. We observed that RGFP966 and PCI34051 increased DR4 expression after incubation on DLD-1 cells, while RGFP966 induced more DR5 expression on WiDr cells, indicating a different role for DR4 or DR5 in these combinations. At last, we show that combined treatment of RGFP966 with TRAIL variants (rhTRAIL 4C7/DHER) increases apoptosis on 3D tumor spheroid models.

## 1. Introduction

Cancer occurs when cells divide in an uncontrolled fashion and escape the strict mechanisms of cell death. A promising anti-cancer treatment is to induce apoptosis to prevent malignant cells from proliferating. Tumor necrosis factor-related apoptosis inducing ligand (TRAIL) is considered as a remarkable anti-cancer therapeutic as it has the ability to selectively kill tumor cells, but not normal cells [1]. Trimeric recombinant human TRAIL can bind to two death receptors, DR4 (TRAIL-R1) and DR5 (TRAIL-R2) for initiating apoptosis signaling [2,3]. Intracellular death domains in death receptors interact with Fas-associated death domain (FADD) and pro-caspase 8 or 10, together forming the death-inducing signaling complex (DISC) [4,5]. This assembly promotes the activation of caspase 8, which then cleaves the effector caspases, caspase 3 and 7, which ultimately induces DNA fragmentation and executes apoptosis via the extrinsic pathway [6,7]. Activated caspase-8 can also cleave Bid thereby generating truncated Bid (tBid), which interacts with Bax and Bak on the mitochondria and promotes the release of cytochrome C. This enzyme together with Apaf-1 and caspase 9 forms a functional apoptosome and results in apoptosis via the intrinsic pathway [8,9].

A recombinant human soluble protein corresponding to 114–281 amino acids of TRAIL has been developed as a clinical anti-cancer drug Dulanermin. Early clinical Phase I study showed that Dulanermin was safe in patients with advanced cancer. In addition, peak concentration in serum in a dose-escalation study was equivalent to those associated with preclinical antitumor efficacy. However, only 2 patients (3%) with chondrosarcoma had partial treatment responses longer than 6 months [10]. This may be related to resistance to TRAIL, but the resistance mechanism is quite complex and may involve multiple epigenetic alterations.

With the development of Chromatin Immunoprecipitation Sequencing (ChIP-Seq) it has now been widely accepted that changes of epigenetic modifications can attribute to tumor progression, drug resistance, or immune tolerance [11,12,13]. These changes are independent of DNA sequence alterations and include at least four modifications of DNA and sixteen classes of histone modifications [14]. One of the important histone modifications is the histone acetylation, which influences a broad range of gene activities, such as chromatin condensation, transcription, DNA repair, and DNA replication. This dynamic process is catalyzed by three groups of enzymes: (1) Histone acetyltransferases (HATs), also known as ‘writers’, are responsible to transfer acetyl groups to targeted lysine residues in the amino-terminal tails of core histone proteins; (2) Histone deacetylases (HDACs), known as ‘erasers’, are found to remove acetyl groups thereby leading to advanced folding of nucleosome to condensed structures; (3) Bromodomain proteins, known as ‘readers’, specifically recognize acetylated lysine residues. Aberrant HATs or HDACs activities have been linked to numerous tumors, such as breast, lung, colorectal, and ovarian cancer. For example, high expression of Class I HDACs has been found to induce cell proliferation in colon tumors, including HT-29, HCT 116, and SW480 cells [15,16,17]. Mutations in the HAT genes CREBBP and EP300 were linked to ovarian, breast, colorectal, and lung tumor types [18,19]. Therefore, inhibition of deacetylation by using HDACi’s seems an attractive strategy for developing anticancer drugs.

Human HDACs are classified into four classes based on their sequence homology to the yeast original enzymes: (i) Class I (HDAC1–3 and HDAC8), (ii) Class II (HDAC4–7, HDAC9, and HDAC10), (iii) Class IV (HDAC11), which are all Zn^2+^ dependent, and (iv) Class III (SIRT1–7), which are all NAD+ dependent [20,21]. US Food and Drug Administration has already approved several broad spectrum HDACi’s, including Belinostat (PXD101), Romidepsin (FK228), and Vorinostat (SAHA) for treating T-cell lymphoma [22,23,24]. Panobinostat (Farydak) is the first approved HDACi for patients with multiple myeloma [25]. However, the European Medicines Agency only approved Farydak as other inhibitors showed rare life-threatening side effects [26]. For example, Panobinostat was found to cause limited hematologic adverse effects in combination with Lenalidomide/Bortezomib/Dexamethasone in Phase I/II trials [27]. However, SAHA was reported to induce pulmonary embolism, deep vein thrombosis, and hyper glycaemia. PXD101 and FK228 cause infections and tumor lysis syndrome [28]. These safety concerns led to great efforts from researchers aiming at development of specific HDACi’s. Currently, a number of HDAC-selective inhibitors are under investigation for use in oncology, including (1) RGFP966, a HDAC3-specific inhibitor, decreases the growth of prostate cancer models [29]; (2) PCI34051, a HDAC8-specific inhibitor, induces apoptosis of T-cell malignancies [30]; and (3) Tubacin, a HDAC6-specific inhibitor, suppresses proliferation of acute lymphoblastic leukemia cells [31]. The mentioned selective HDACi’s are supposed to give lower toxicity than pan-HDACi’s. Therefore, they seem appropriate to be used in combination therapies aimed at improving antitumor effects synergistically. Hence, we chose RGFP966 or PCI34051 to be used in combination with apoptosis-inducing ligands in colon cancer. Both rhTRAIL 4C7 and DHER have shown superior apoptosis-inducing effects in colon tumor cells compared to rhTRAIL, but a number of cell lines to a lesser (DLD-1) or higher (WiDr) extend show resistance [32,33].

Here, we investigated the role of individual HDAC1, 2, and 3 in colon cancer cells DLD-1 and WiDr using different HDACi’s with partially overlapping specificities. Additionally, we combined these inhibitors with two TRAIL variants, DR4-specific rhTRAIL 4C7 and DR5-specific rhTRAIL DHER, to further unravel the antitumor effects. Our results show that RGFP966 improves TRAIL-induced apoptosis via both DR4 and DR5 receptors and its antitumor effect in combination with rhTRAIL 4C7 or DHER is close to the effect by SAHA combined with TRAIL variants. PCI34051 also enhances cell death in combination with rhTRAIL variants. Moreover, the same trend can be found on *HDAC1*, *2*, *3*, or *8* knocked down cell lines. Finally, we measured the antitumor effect in 3D spheroid culture mimicking in vivo models. In trying to understand mechanism, we monitored the surface expression of DR4 and DR5 and we analyzed cell cycle changes in HDACi’s-treated cells.

## 2. Results

### 2.1. HDACi’s Enhance Cell Death in Combination with Receptor-Specific TRAIL Variants rhTRAIL 4C7 and DHER

**Figure 1:**
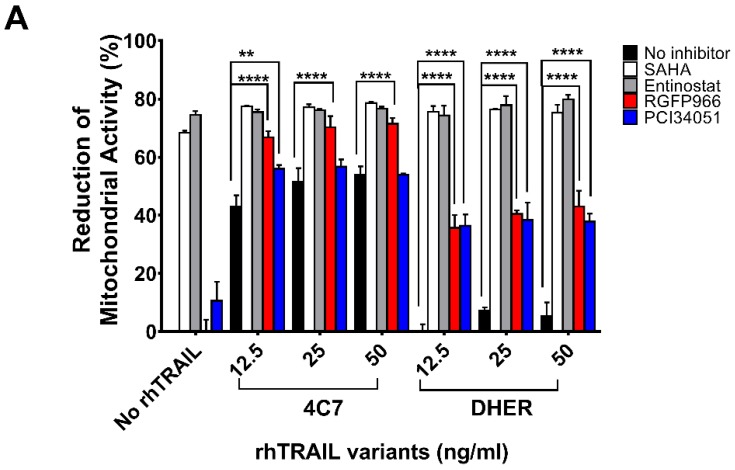

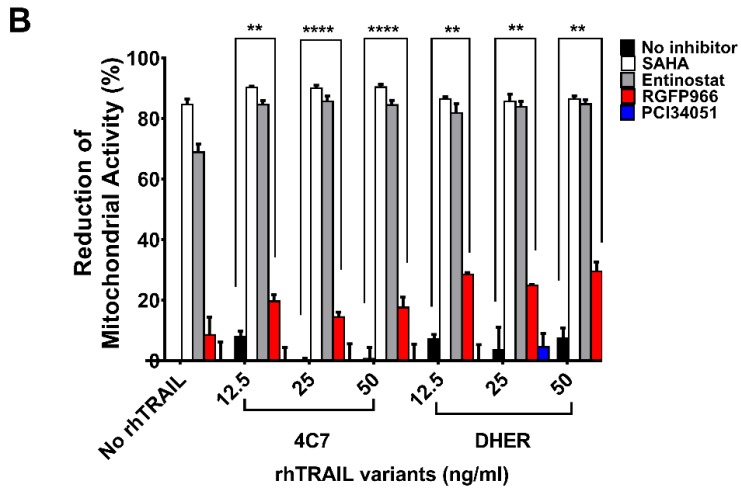
Alterations of cell viability after treatment with HDAC inhibitors and rhTRAIL variants 4C7 or DHER. DLD-1 cells (**A**) or WiDr cells (**B**) were firstly treated with 10 μM HDAC inhibitors including SAHA, Entinostat, RGFP966, or PCI34051, respectively, for 24 h and the day after cells were incubated with rhTRAIL 4C7 or DHER overnight. Cell viability was determined by MTS assay. The values shown are mean ± SD from one of three experiments performed in triplicate. *p* values were analyzed by one-way ANOVA in Turkey’s multiple comparison with Graphpad Prism version 7.0. ** 0.001 ≤ *p* ≤ 0.01, **** *p* ≤ 0.0001.

It has been found that HDAC1, 2, 3, and 8 are overexpressed in colon tumor cells, but the function of individual HDAC in cancer metabolism is still unclear. To study the role of the respective HDACs we performed cell viability assays testing the sensitivities of DLD-1 and WiDr cells to various HDACi’s. Additionally, we used DR4-specific TRAIL variant 4C7 and DR5-specific TRAIL variant DHER to study apoptosis via DR4 and DR5 separately. Here, we observed that single treatment of SAHA already induces a relatively high cell death (Figure 1A,B). To focus on Class I HDACs, we chose (i) Entinostat, a HDAC1, 2, 3 selective inhibitor; (ii) RGFP966, a HDAC3-specific inhibitor; and (iii) PCI34051, a HDAC8-specific inhibitor. Figure 1A shows that at 10 μM Entinostat on its own induces around 70% cell death whereas RGFP966 or PCI34051 does not cause cell death. However, RGFP966 significantly increases cell death in the presence of either rhTRAIL 4C7 or DHER in DLD-1 cells indicating that HDAC3 may play an important role in stimulating TRAIL-induced cell death. Additionally, we detected additive cell death caused by PCI34051+rhTRAIL 4C7 or DHER in DLD-1 cells, which indicates that PCI34051 may trigger cell death dependent of TRAIL-induced pathways. Since DLD-1 cells are sensitive to rhTRAIL 4C7, the absolute increase in dead cells caused by rhTRAIL 4C7+RGFP966/PCI34051 is less pronounced than by rhTRAIL DHER+RGFP966/PCI34051 (Figure 1A). Interestingly, in WiDr cells rhTRAIL DHER+RGFP966 induces more cell death than rhTRAIL 4C7+RGFP966 (Figure 1B). This implies that DR5 may be more active than DR4 in TRAIL-mediated apoptotic signaling treated by the combination. A relative higher increase of cell death was detected at a low concentration (5 µM) of HDACi’s (Figure S1). Notably, we are the first to show that RGFP966/PCI34051 enhances TRAIL sensitivity in colon cancer cells. The DLD-1 cell death caused by RGFP966+rhTRAIL 4C7 is quite comparable to the one caused by SAHA/Entinostat+rhTRAIL 4C7 treatment, which implies a crucial role for HDAC3 in enhancing TRAIL-mediated cell death.

### 2.2. RGFP966 and PCI34051 Improve TRAIL-Induced Apoptosis

**Figure 2:**
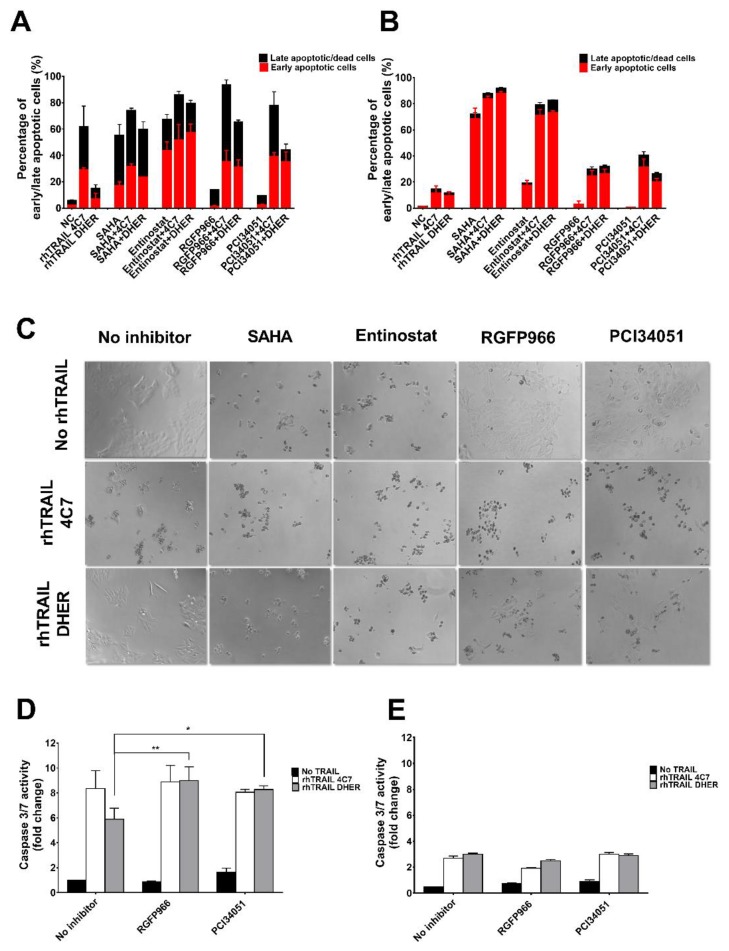
HDAC3-specific inhibitor RGFP966 or HDAC8-specific inhibitor PCI34051 increases TRAIL-mediated apoptosis. DLD-1 (**A**) or WiDr (**B**) cells were pre-treated with 5 μM HDAC inhibitors for 24 h and then incubated with 25 ng/mL rhTRAIL 4C7 or DHER for overnight. Early or late apoptotic cells were detected using the Violet Ratiometric Membrane Asymmetry Probe. NC represents the cells without treatment with HDAC inhibitors or rhTRAIL variants. Statistics: Entinostat vs. Entinostat+TRAIL variants, 0.0001 ≤ *p* ≤ 0.001; TRAIL variants vs. Entinostat+TRAIL variants, 0.0001 ≤ *p* ≤ 0.001 (**C**) Morphological changes of DLD-1 cells treated with HDAC inhibitors and rhTRAIL 4C7 or DHER observed under an inverted light microscope with 20× magnification. DLD-1 (**D**) or WiDr (**E**) cells were pre-treated by 10 μM RGFP966 or PCI34051 for 24 h and stimulated with 25 ng/mL rhTRAIL 4C7 or DHER overnight. Caspase 3/7 activity was measured after 2 h incubation. The values shown are mean ± SD from one of three experiments performed in triplicate. *p* values were analyzed by one-way ANOVA in Turkey’s multiple comparison with Graphpad Prism version 7.0. * 0.01 ≤ *p* ≤ 0.05, ** 0.001 ≤ *p* ≤ 0.01.

In order to further investigate the mode of action of the combination treatment of HDACi and rhTRAIL variants on colon cancer cells, we examined apoptotic cells using the Violet Ratiometric Membrane Asymmetry Probe. Figure 2A shows that incubation by 5 µM SAHA or Entinostat on DLD-1 already induced early and late apoptosis whereas a combination treatment with rhTRAIL 4C7 or DHER further increased apoptosis. This additional effect was less pronounced using rhTRAIL 4C7 than DHER, which is due to the already high sensitivity of DLD-1 cells to rhTRAIL 4C7. Different from DLD-1 cells, WiDr cells are resistant to both rhTRAIL 4C7 and DHER (Figure 2B). Entinostat in combination with any of the TRAIL variants significantly enhances cell apoptosis in WiDr cells. In line with the cell viability study, 5 or 10 µM RGFP966 or PCI34051 in combination with rhTRAIL 4C7 or DHER largely increases apoptosis in DLD-1 and WiDr cells (Figure 2A and Figure S2B). Additionally, microscopic examination of DLD-1 cells (Figure 2C) or WiDr cells (Figure S2A), reveals that cells detach, lose confluent growth, and start floating in the medium, which is triggered by the combination treatment. Furthermore, caspase-3/7 activity was found to be increased by combination treatment on DLD-1 cells indicating that caspase-dependent apoptosis is transmitted downstream pathways (Figure 2D,E). In conclusion, these results confirm that RGFP966 and PCI34051 sensitize TRAIL-induced apoptosis via DR4 and DR5 on DLD-1 cells, while this increased apoptosis is less pronounced on TRAIL-resistant WiDr cells. However, Entinostat significantly increased DR4 and DR5-mediated apoptosis on WiDr cells, indicating that HDAC1 and HDAC2 may be crucial for sensitizing TRAIL-resistant cells to TRAIL.

### 2.3. Knockdown of HDAC1, 2, 3, 8 Enhances TRAIL Sensitivity

**Figure 3:**
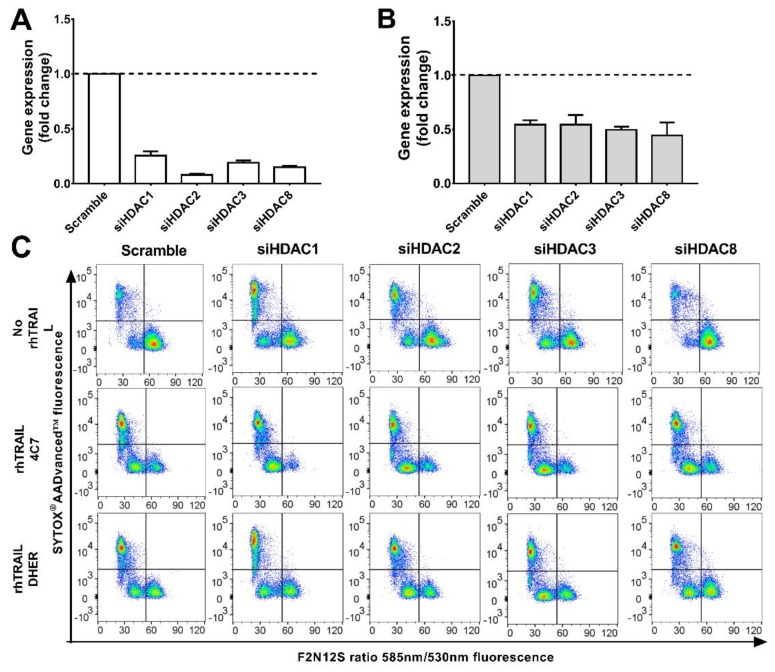

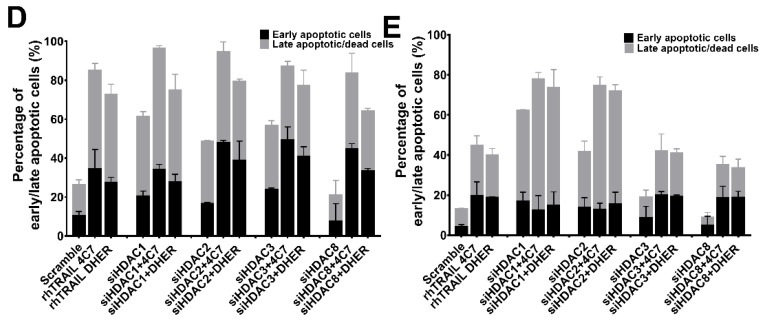
Knockdown of *HDAC1*, *2*, *3*, or *8* enhances TRAIL sensitivity. DLD-1 (**A**) or WiDr (**B**) cells were transfected with siRNAs of scramble or *HDAC1*, *2*, *3*, and *8* respectively for 72 h using Lipofectamine 2000. Relative mRNA levels were normalized by *α-tubulin*. The values shown are mean ± SD from one of three experiments performed in triplicate. (**C**) After 72 h transfection, DLD-1 cells were treated with 25 ng/mL rhTRAIL 4C7 or DHER and apoptotic cells were detected using the Violet Ratiometric Membrane Asymmetry Probe. The lower right group represents living cells, the lower left group represents early apoptotic cells, and the upper left group represents late apoptotic or dead cells. Statistical analysis of DLD-1 (**D**) or WiDr (**E**) cells was shown.

The above results indicate that inhibition of HDAC1, 2, 3, and 8 increases the sensitivity to TRAIL-induced apoptosis. Therefore, we next focus on silencing *HDAC1*, *2*, *3*, and *8* individually and investigating the alterations of colon cancer cells (DLD-1 and WiDr) in response to DR4 and DR5-induced apoptosis. Cells were transduced either with scrambled siRNAs or a pool of *HDAC1*, *2*, *3*, or *8* siRNAs. qRT-PCR shows a clear decrease in expression of *HDAC1*, *2*, *3*, and *8* at mRNA level with knockdown levels in DLD-1 being better than in WiDr (Figure 3A,B). Apoptotic cells induced by knockdown of HDAC genes alone or in combination with rhTRAIL 4C7 or DHER treatment were investigated. It can be seen that downregulating the expression of HDAC1, 2, or 3 on DLD-1 (Figure 3C) induces early and late apoptosis, while HDAC 8 does not. This implies that HDAC1, 2, and 3 are connected with cell apoptosis and therefore may crosstalk with a TRAIL-mediated apoptosis pathway. Interestingly, early apoptotic cells increased after adding rhTRAIL 4C7 or DHER on *HDAC* knockdown cells in comparison with scramble control as shown in Figure 3D,E, which indicates that both DR4 and DR5 are involved in the signaling pathway. Notably, the amount of apoptotic cells induced by rhTRAIL variants in *HDAC3* or *8* knockdown DLD-1 cells are almost the same as in *HDAC1* or *2* knockdown cells. In WiDr cells, downregulation of HDAC1 or 2 induces apoptosis, while HDAC3 or 8 not, which is in line with the observed low toxicity of HDAC3- and 8-specific inhibitors (Figure 3E and Figure S3). Moreover, percentages of apoptotic cells induced by rhTRAIL variants in *HDAC3* or *8* knockdown WiDr cells are lower than that in *HDAC1* or *2* knockdown cells, implying an important role of HDAC1 or 2 in connection to TRAIL-mediated apoptosis.

### 2.4. RGFP966 or PCI34051 Improves TRAIL-Induced Apoptosis in 3D Spheroid Model

**Figure 4:**
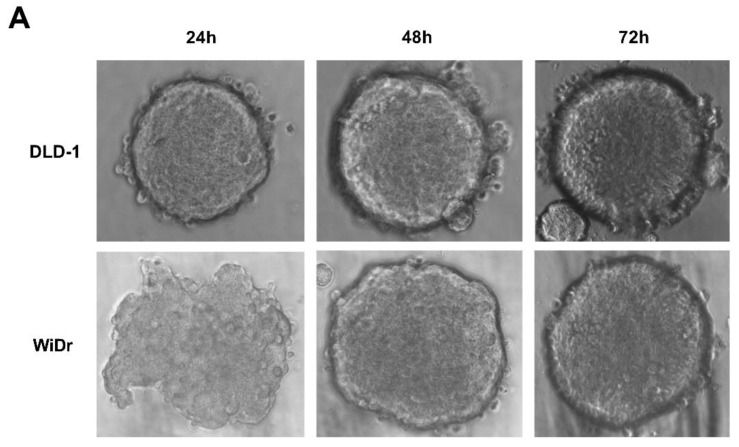

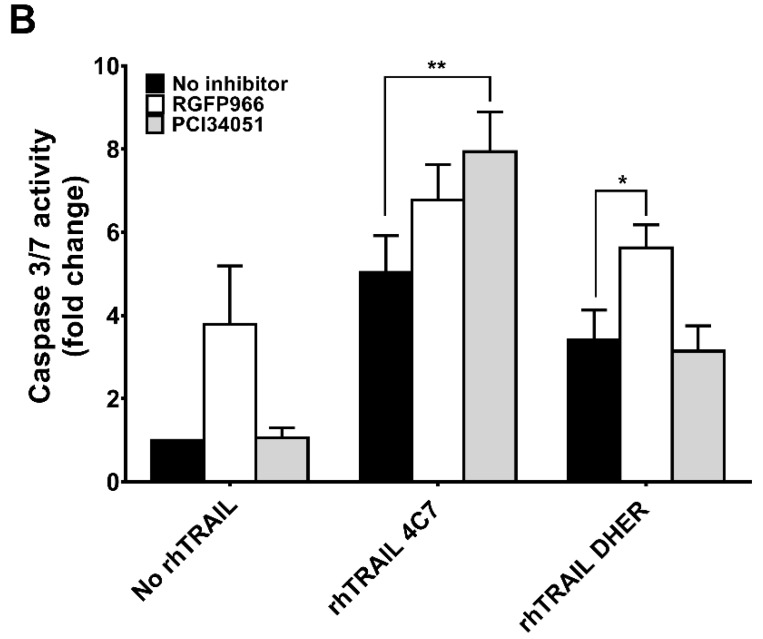
RGFP966 or PCI34051 enhances TRAIL sensitivity on 3D spheroid model. (**A**) 3D spheroids were constructed on DLD-1 or WiDr cells after 72 h culturing in ultra-low attachment round bottom plates. Morphology changes were observed under an inverted light microscope with 40× magnification. (**B**) The DLD-1 spheroids were generated and transferred to a 96-well white wall plate and caspase3/7 activity was detected after adding 5 ng/mL rhTRAIL 4C7 or DHER overnight. Luminescence were measured after 2 h incubation with caspase 3/7 reagent. *p* values were analyzed by one-way ANOVA in Turkey’s multiple comparison with Graphpad Prism version 7.0. * 0.01 ≤ *p* ≤ 0.05, ** 0.001 ≤ *p* ≤ 0.01.

As we concluded above, RGFP966 and PCI34051 are promising drugs in combination with rhTRAIL 4C7 or DHER to improve apoptosis in colon cancer cells. To mimic actual tumor microenvironment and further demonstrate feasibility of our study, we investigated this combination on 3D spheroids which are considered as more valid models to recapitulate features of tumor micro metastases as they have a specific architecture that 2D monolayer culture cannot produce. We firstly established 3D spheroids by culturing DLD-1 and WiDr cells in ultra-low attachment plates (Figure 4A). Spheroids were subsequently treated with RGFP966 or PCI34051+rhTRAIL 4C7 or DHER followed by caspase 3/7 activity assay. Active caspase-3/7 largely increased after adding rhTRAIL 4C7 or DHER in comparison with only RGFP966 incubation. Additionally, activity of caspase-3/7 also improved after incubating with rhTRAIL 4C7 and PCI34051, while no obvious activity increase was detected after incubating with rhTRAIL DHER and PCI34051. 

### 2.5. Expressions of Death Receptors and Cell Cycle Alter Upon HDACi’s Treatment

**Figure 5:**
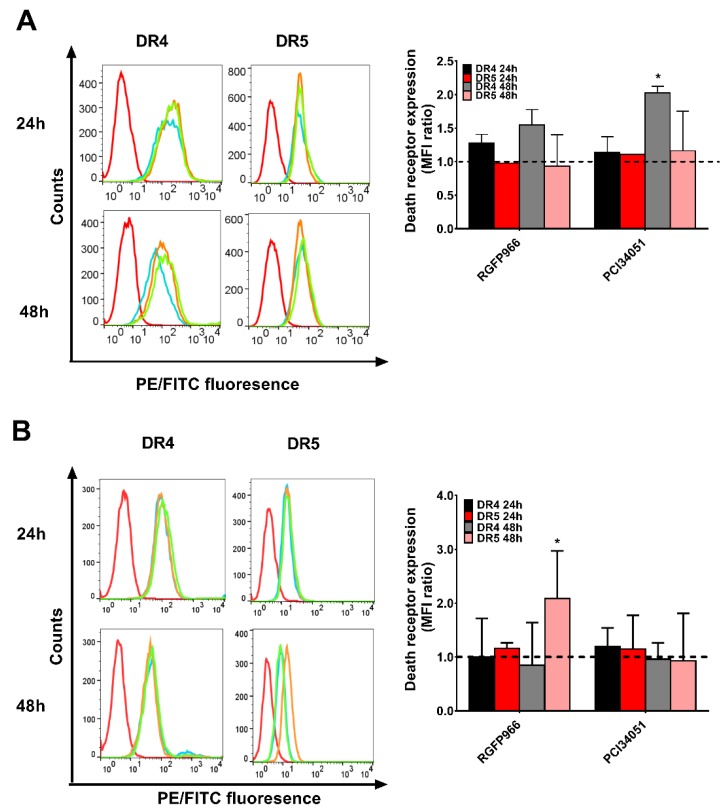
RGFP966 and PCI34051 changes expression of DR4 or DR5. DLD-1 (**A**) or WiDr (**B**) cells were incubated by RGFP966 or PCI34051 for 24 h or 48 h, and DR4 and DR5 expression were measured after incubation. The left panel shows the fluorescence shift and the right panel shows the geometric mean changes relative to untreated cells (dash line). On the left panel, red open lines show background signal of PE or FITC by adding only secondary antibodies to the cells. Blue open lines represent cells without HDAC inhibitor treatment, orange lines represent cells treated with 5 μM RGFP966 and green open lines represent cells treated with 5 μM PCI34051. *p* values were analyzed by one-way ANOVA in Turkey’s multiple comparison with Graphpad Prism version 7.0. * 0.01 ≤ *p* ≤ 0.05.

**Figure 6:**
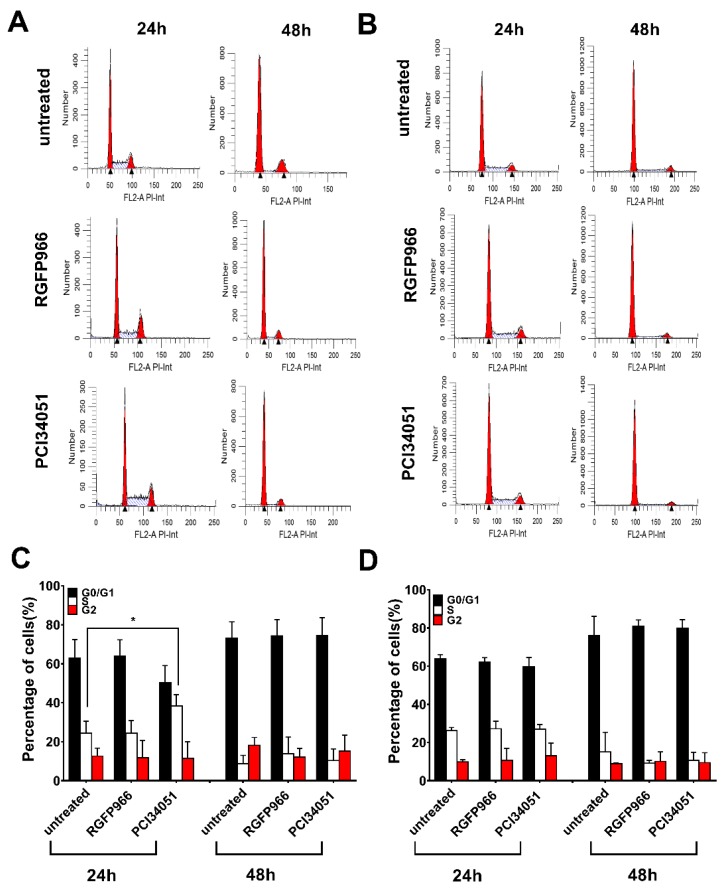
PCI34051 arrest in the G0/G1 phase in DLD-1 cells after 24 h incubation. DLD-1 (**A**,**C**) or WiDr (**B**,**D**) cells were incubated with 5 μM RGFP966 or PCI34051 for 24 h or 48 h and then cell cycles were measured after incubation. Statistical analysis in (**C**) or (**D**) are according to (**A**) or (**B**). The values shown are mean ± SD from one of three experiments performed in triplicate. *p* values were analyzed by one-way ANOVA in Turkey’s multiple comparison with Graphpad Prism version 7.0. * 0.01 ≤ *p* ≤ 0.05.

It has been discovered that SAHA induces overexpression of DR5 on the hepatocellular carcinoma cell membrane leading to improvement of TRAIL sensitivity [34]. However, whether this mechanism happens in colon cancer cells is still unclear. To further study the mechanism, we investigated the expression of both DR4 and DR5 after incubation for 48 h with different concentration of RGFP966 or PCI34051 in a 2D culture. Surprisingly, we detected a 1.5-fold increase of DR4 but not DR5 after incubating with RGFP966 for 48 h and a 2-fold increase of DR4 after incubating with PCI34051 on DLD-1 cells (Figure 5A and Figure S4A). Interestingly, DR5 expression increased after incubating with RGFP966 on WiDr cells for 48 h (Figure 5B and Figure S4B). These changes of death receptor expression may lead to a different sensitivity to TRAIL-mediated apoptosis. In addition, we also studied alterations of the cell cycle with treatment of HDACi’s as apoptosis is related to cell cycle arrest [35]. Figure 6A,C showed that PCI34051 arrests G0/G1 phase after incubating 24 h, while RGFP966 does not change the cell cycle in DLD-1 cell line. Either RGFP966 or PCI34051 changes the cell cycle in WiDr cell line (Figure 6B,D). It has been reported that SAHA and Entinostat induced cell cycle arrest. We also observed G2 phase arrest after incubating with SAHA for 24 h and G0/G1 phase arrest after incubating with Entinostat for 24 h in DLD-1 and WiDr cells (Figure S5). Alterations of the cell cycle may contribute to the mechanism of increasing TRAIL sensitivity as well.

## 3. Discussion

Administration of TRAIL has been considered as a promising antitumor therapy for a long time due to its tumor selective properties. However, studies have shown that approximately 50% of the colorectal cancer cells are resistant to TRAIL [36,37], seemingly due to various genetic and epigenetic modifications in the signaling pathway. Combination therapies aimed at relieving this resistance have recently been investigated. In clinical studies, combinations of Dulanermin with FOLFIRI regimen (with or without Bevacizumab)/Cetuximab/Irinotecan have already been tested but without the desired result (NCT00671372, NCT00873756) implying that is still a need for a good combination drug for Dulanermin. Here, we for the first time evidence that combining RGFP966 or PCI34051 with our mutants rhTRAIL 4C7 or DHER significantly improves apoptosis induction [32,33].

Many studies have discussed the complexity of the mechanism of TRAIL resistance and one of elements is epigenetic regulation such as histone acetylation [38,39]. In previous studies, TRAIL has been combined with SAHA in hepatocellular carcinoma, non-small cell lung cancer, or breast cancer and this showed a large improvement in apoptosis indicating the potential role of epigenetic modification [34,40,41]. Since SAHA as a broad-spectrum inhibitor has many side effects for clinical use, we tested different small spectrum HDACi’s for their potential to enhance TRAIL mediated apoptosis in two colon cancer cells DLD-1 and WiDr. Notably, our MTS results show that HDAC3-specific inhibitor RGFP966 and HDAC8-specific inhibitor PCI34051 both increase cell death of DLD-1 and WiDr after treatment with rhTRAIL 4C7 or DHER. Further investigations of cell apoptosis suggest that inhibition of HDAC3 or 8 facilitate rhTRAIL 4C7- or DHER-induced apoptosis.

We next generated *HDAC1*, *2*, *3*, and *8* knockdown cells for more precise mechanism study. Studies have shown roles of HDAC3 or HDAC8 in colon cancer cells. Spurling et al. reported that HDAC3 is overexpressed in SW480 cells resulting in proliferation and differentiation [16]. Kang et al. recently found that HDAC8 is associated to activator of transcription 3 (STAT3)/specificity transcription 3 (Sp3) exchange and induces Bmf-dependent apoptosis [32]. DLD-1 and WiDr cells response differently on the administration of HDAC inhibition, which may be related to the differences in epigenetic and genetic background of these two cell lines. It has been reported that DLD-1 cells contain a *KRAS* mutation while WiDr does not [42,43]. This mutation may be connected to hyperactivation of DNA methylation in these colon cancer cells since upregulation of DNA methylation was discovered after transformation of fibroblasts by *ras* oncogene [16]. Increasing DNA methylation may lead to recruitment of HDAC explaining a more pronounced effect of HDACi’s in DLD-1 cells [44]. Three-dimensional spheroid models have been developed to mimic actual tumor microenvironment in the body. Here, we generated 3D spheroids and showed increased apoptosis induced by RGFP966 in combination with rhTRAIL 4C7 or DHER. There are some studies show that TRAIL-induced apoptosis is regulated by post-translational modifications of death receptors [45]. O-glycosylation of DR4 and DR5 was proven to control the sensitivity of many cancer cells to TRAIL [36]. Subsequently, Dufour et al. reported that N-glycosylated DR4 promotes TRAIL signaling [46]. Moreover, we previously found that DR5 is activated by fucosylation for TRAIL-induced apoptosis using our TRAIL variants [47]. Interestingly, recently a relation between HDAC inhibition and glycosylation pattern was reported which can hint at an explanation for increasing sensitivity of TRAIL receptors in the presence of HDAC inhibitors [48]. In our current study, we investigated the expression of DR4 and DR5 on the cell membrane and our results show significant DR4 but not DR5 expression enhancement on DLD-1 cells, while DR5 but not DR4 expression is increased on WiDr cells. It is of interest to further explore precise mechanisms of alterations of DR4 and DR5 expression induced by HDACi’s using ChIP-Seq. Interestingly, a study showed that G1 cell cycle arrest in melanoma cells is strongly correlated with enhanced TRAIL-mediated apoptosis [35]. We observed G0/G1 arrest upon the treatment of PCI34051 indicating that a similar mechanism occurs in colon cancer cells. Taken together, our findings give a new insight into the effect of HDACs on TRAIL-mediated apoptosis and they imply a promising novel antitumor therapy using TRAIL variants with HDAC3-specific inhibitor RGFP966 or HDAC8-specific inhibitor PCI34051.

## 4. Materials and Methods

### 4.1. Cell Lines and Culture Conditions

Human colon cancer cells of DLD-1 and WiDr were obtained from American Type Culture Collection (ATCC, Wesel, Germany) and cultured in RPMI1640 medium supplemented with 10% fetal bovine serum (FBS), 100 units/mL Penicillin, and 100 μg/mL Streptomycin in a humidified incubator at 37 °C with 5% carbon dioxide. Basal medium RPMI1640, FBS, Penicillin, and Streptomycin were purchased from Thermo Fisher Scientific (Waltham, MA, USA).

### 4.2. Cell Viability Assay

DR4-specific TRAIL variant rhTRAIL 4C7 and DR5-specific TRAIL variant rhTRAIL DHER (amino acids 114–281) were constructed and produced as previously described [32,33]. Cells were seeded in triplicate in 96-well plates at a concentration of 30,000 cells/mL in 100 μL complete medium and maintained overnight prior to the treatment. Cells were treated with 5 μM or 10 μM HDACi’s including SAHA, Entinostat, RGFP966, and PCI34051 overnight and 0–50 ng/mL rhTRAIL 4C7 or DHER were added at the following day. After overnight incubation, cells were incubated with 20μL/well MTS reagent (Promega, Madison, WI, USA) for 1.5 h according to the manufacturer’s instruction. Cell viability was determined by measuring the absorbance at 490 nm using a microplate reader (BMG LABTECH, De Meern, Utrecht, The Netherlands). All HDACi’s were purchased from Selleckchem (Munich, Germany).

### 4.3. Apoptotic Assay

The 2 × 10^5^ cells were seeded in 3 mL complete medium in 6-well plates 24 h prior to the treatment. The next day, cells were treated with 10 μM HDACi’s at a final volume of 1 ml overnight. 25 ng/mL rhTRAIL 4C7 or DHER were added at the following day and incubated overnight. After treatment, cells were collected and washed with PBS twice. Cell pellets were resuspended in 200 μL PBS containing reagent A and B from cell apoptotic kit (Violet Ratiometric Membrane Asymmetry Probe/Dead cell Apoptotic Kit) bought from Thermofisher Scientific (Waltham, MA, USA). Cells were measured and analyzed by LSR-II (BD Bioscience, Franklin Lakes, NJ, USA). Imagines were taken under the microscope after treatment at different time points. For HDAC knockdown cells, rhTRAIL 4C7 or DHER were added after 72 h transfection.

### 4.4. Caspase 3/7 Activity Assay

Cells were seeded in triplicated in a 96-well plate with white walls at a density of 30,000 cells/mL overnight before the treatment. Cells were pre-incubated for 24 h with 10 μM HDACi’s and followed by the treatment with 25 ng/mL rhTRAIL 4C7 or DHER. The next day 100 μL reagent was added according to the manufacturer’s protocol and incubated for 2 h at room temperature (Promega, Madison, WI, USA). Luminescent was measured with a Synergy™ H1 plate reader (BioTek, Winooski, VT, USA). For the 3D spheroids, every spheroid was transferred to 96-well plate with white walls before adding reagent. Reagent were incubated for 2 h at room temperature with spheroid and the same measurement as monolayer cells was conducted.

### 4.5. HDAC1, 2, 3, and 8 Knockdown Using siRNA

Cells were seeded at 2 × 10^5^ per well in 6-well plates and incubated for 24 h. The next day cells were transfected with predesigned pool of small interfering RNA (siRNA) oligonucleotides at a final concentration of 600 ng/mL (*HDAC1* and *2*) or 5 nmol/L (*HDAC3* and *8*) with Lipofectamine 2000 Reagent (Thermo Fisher Scientific, Waltham, MA, USA). After 72 h incubation, cells were collected for qRT-PCR. siRNA of *HDAC1* (MISSION, esiRNA HDAC1) and *2* (MISSION, esiRNA HDAC2) were purchased from Millipore Sigma (Burlington, MA, USA). siRNA of *HDAC3* (M-003496-02-0005, siGENOME Human HDAC3 (8841) siRNA-SMART pool) and *8* (M-003500-02-0005, siGENOME Human HDAC8 (55869)-SMART pool) were purchased from GE Healthcare Dharmacon (Lafayette, LA, USA).

### 4.6. 3D Spheroid Construction

1000 cells per well were seeded in ultra-low attachment plates with 96-wells (Corning Incorporated, Kennebunk, ME, USA). Plates were centrifuged at 1000 rpm for 5 min to initiate the formation of 3D spheroid. After 3 days incubation, spheroids were generated and ready for performing experiments.

### 4.7. RNA Isolation and Quantitative Reverse Transcriptase PCR (qRT-PCR)

Cells were washed by PBS and harvested by trypsin. RNA was isolated using Maxwell LEV simply RNA Cells/Tissue Kit (Promega, Madison, WI, USA) and then concentrations of RNA was measured by NanoDrop (Thermo Fisher Scientific, Waltham, MA, USA). cDNA was synthesized from 200 ng RNA using Reverse Transcription Kit (Promega, Madison, WI, USA) according to instruction of manufacture. The 20 ng cDNA and SensiMix SYBRkit (Bioline, Taunton, MA, USA) were used to perform qRT-PCR in an ABI Prism 7900HT Sequence Detection System (Thermo Fisher Scientific, Waltham, MA, USA). Primers sets are listed in Table S1. Data was analyzed by SDS v.2.3 software (Applied Biosystems, Foster City, CA, USA). mRNA level of α-tublin was measured and used as a reference for data normalization.

### 4.8. Death Receptor Expression Analysis

Cells were seeded at 2 × 10^5^ per well in 6-well plate overnight. The next day, 5 μM or 10 μM HDACi’s were added and incubated for 24 h or 48 h. After incubation, cells were collected and washed with FACS buffer (PBS with 1% FBS). Then cells were incubated with primary antibodies for DR4 (abcam, Cambridge, UK) or DR5 (EXBIO Praha, Nad Safinou, Czech Republic) on ice for 1 h. Subsequently, cells were washed and incubated with R-Rhycoerythrin (PE) conjugated goat anti-rabbit antibody (Southern Biotech, Birmingham, AL, USA) or Fluorescein (FITC) conjugated donkey anti-mouse antibody (Jackson ImmunoResearch Europe, Cambridge, UK) on ice for 1 h. DR4 and DR5 expression was detected using a FACS Calibur flow cytometer (BD Bioscience, Franklin Lakes, NJ, USA).

### 4.9. Cell Cycle Analysis

Cells were seeded at 2 × 10^5^ per well in 6-well plate overnight. The following day cells were treated with 5 μM HDACi’s for 24 h or 48 h. After incubation cells were harvested by PBS and trypsin. Cell pellets were washed and cells were fixed using cold 70% ethanol overnight at 4 degree. At last, cells were harvested and DNA was stained with 20 ug/mL propidium iodide (Thermo Fisher Scientific, Waltham, MA, USA). Cell cycle were detected using a FACS Calibur flow cytometer (BD Bioscience, Franklin Lakes, NJ, USA).

### 4.10. Data Analysis

Data were presented as mean ± SD from one of three experiments performed in triplicates. *p* values were analyzed by one-way ANOVA in Turkey’s multiple comparison with Graphpad Prism version 7.0 (San Diego, CA, USA). ** *p* ≤ 0.005, *** *p* ≤ 0.0005, **** *p* ≤ 0.0001. Cell apoptosis and death receptor expression were analyzed by FlowJo V10 (BD Bioscience, Franklin Lakes, NJ, USA). Cell cycle was analyzed by ModFit LT (Verity Software House, Topsham, ME, USA).

## 5. Conclusions

Our data show that both HDAC3 inhibitor (RGFP966) and HDAC8 inhibitor (PCI34051) sensitize to TRAIL-mediated apoptosis most likely through changes in death receptor expression and cell cycle arrest. This represents a novel effective combination therapy to kill colon cancer cells.

## Supplementary Materials

The following are available online at https://www.mdpi.com/2072-6694/11/5/645/s1, Figure S1: 5 μM of HDAC inhibitors RGFP966 or PCI34051 increase the sensitivity of DLD-1 (A) or WiDr (B) cells to rhTRAIL 4C7 or DHER, Figure S2: HDAC inhibitors increase TRAIL-mediated apoptosis, Figure S3: After 72 h transfection with siRNA, WiDr cells were treated with 25 ng/mL rhTRAIL 4C7 or DHER and apoptotic cells were detected using the Violet Ratiometric Membrane Asymmetry Probe, Figure S4: Alterations of death receptor expression after incubating with 10 μM RGFP966 or PCI34051 for 24 h or 48 h on DLD-1 (A) or WiDr (B) cells, Figure S5: SAHA or Entinostat changes cell cycle after incubating for 24 h on DLD-1 (A) or WiDr (B) cells. Red peaks represent G0/G1 and G2 phase, Table S1: List of primer sets used for qRT-PCR.
