# Supplementary material for: Histone Deacetylase Inhibitors Sensitize TRAIL-Induced Apoptosis in Colon Cancer Cells
Source: Cancers (Basel). 2019 May 10;11(5):645. doi: 10.3390/cancers11050645 (PMC6562715; doi:10.3390/cancers11050645)
Supplement: Supplementary file 1 [file cancers-11-00645-s001.pdf]

Supplementary Material

Histone Deacetylase Inhibitors Sensitize TRAIL-Induced Apoptosis in Colon Cancer Cells

Baojie Zhang, Bin Liu, Deng Chen, Rita Setroikromo, Hidde J. Haisma and Wim J. Quax

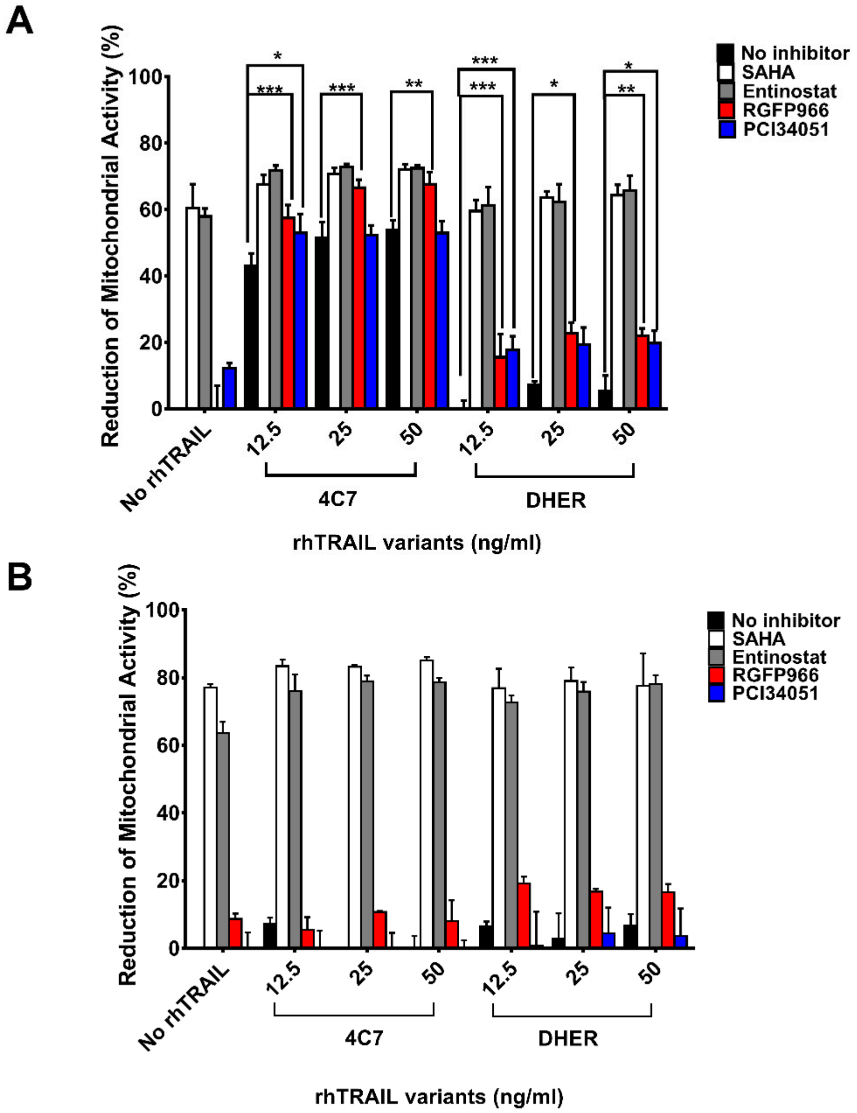

Figure S1. 5 μM of HDAC inhibitors RGFP966 or PCI34051 increase the sensitivity of DLD-1 (**A**) or WiDr (**B**) cells to rhTRAIL 4C7 or DHER. The values shown are mean ± SD from one of three experiments performed in triplicate. p values were analyzed by one-way ANOVA in Turkey's multiple comparison with Graphpad Prism version 7.0. * $0.01 \le p \le 0.05$, ** $0.001 \le p \le 0.01$, *** $0.0001 \le p \le 0.001$.

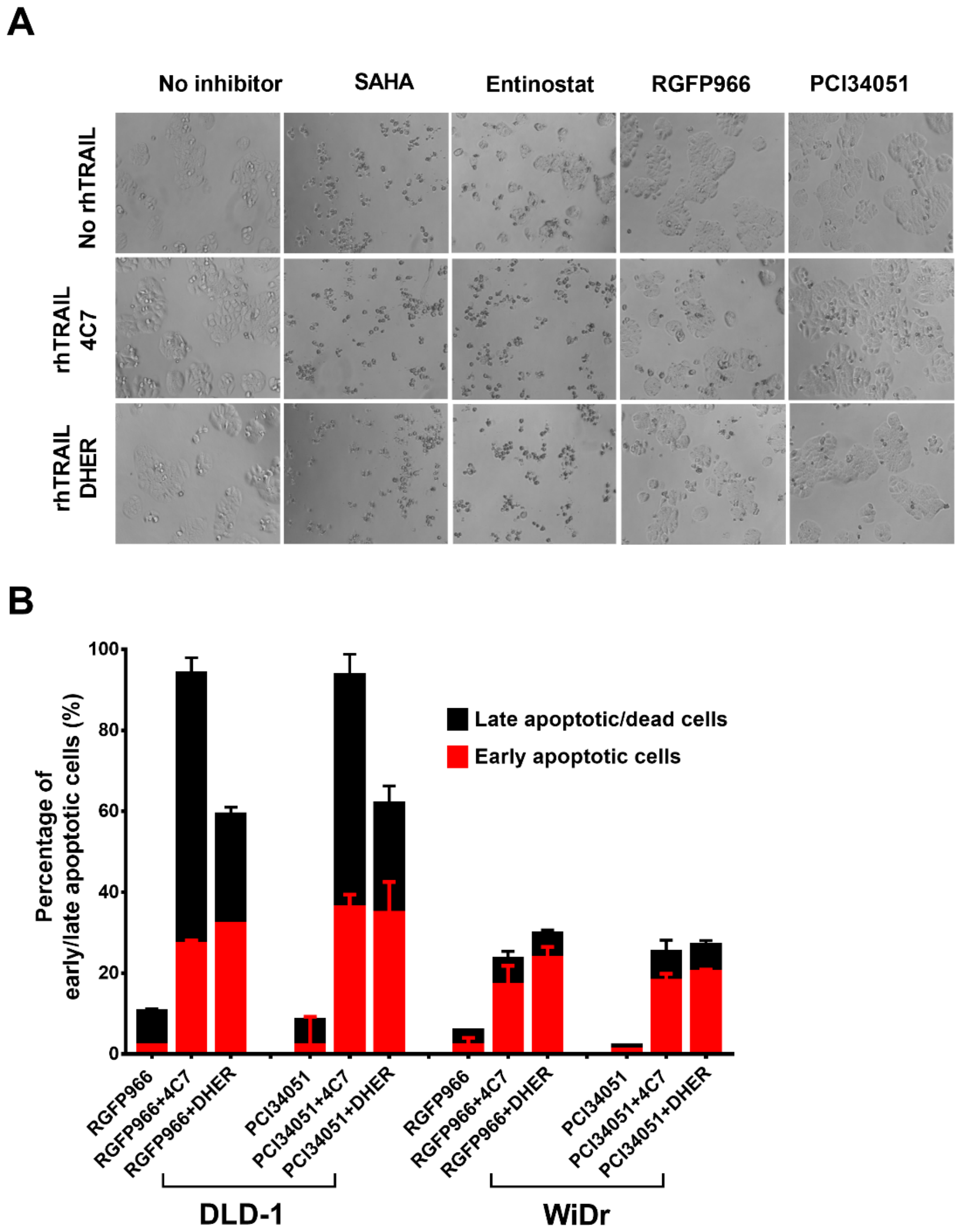

Figure S2. HDAC inhibitors increase TRAIL-mediated apoptosis. (**A**) Morphological changes of WiDr cells treated with 5 μM HDAC inhibitors and 25 ng/mL rhTRAIL 4C7 or DHER observed under an inverted light microscope with 20× magnification. (**B**) DLD-1 or WiDr cells were pre-treated by 10 μM HDAC inhibitors for 24 h and then incubate with 25 ng/mL rhTRAIL 4C7 or DHER for overnight. Apoptotic cells were detected using the Violet Ratiometric Membrane Asymmetry Probe. The values shown are mean ± SD from one of three experiments performed in triplicate.

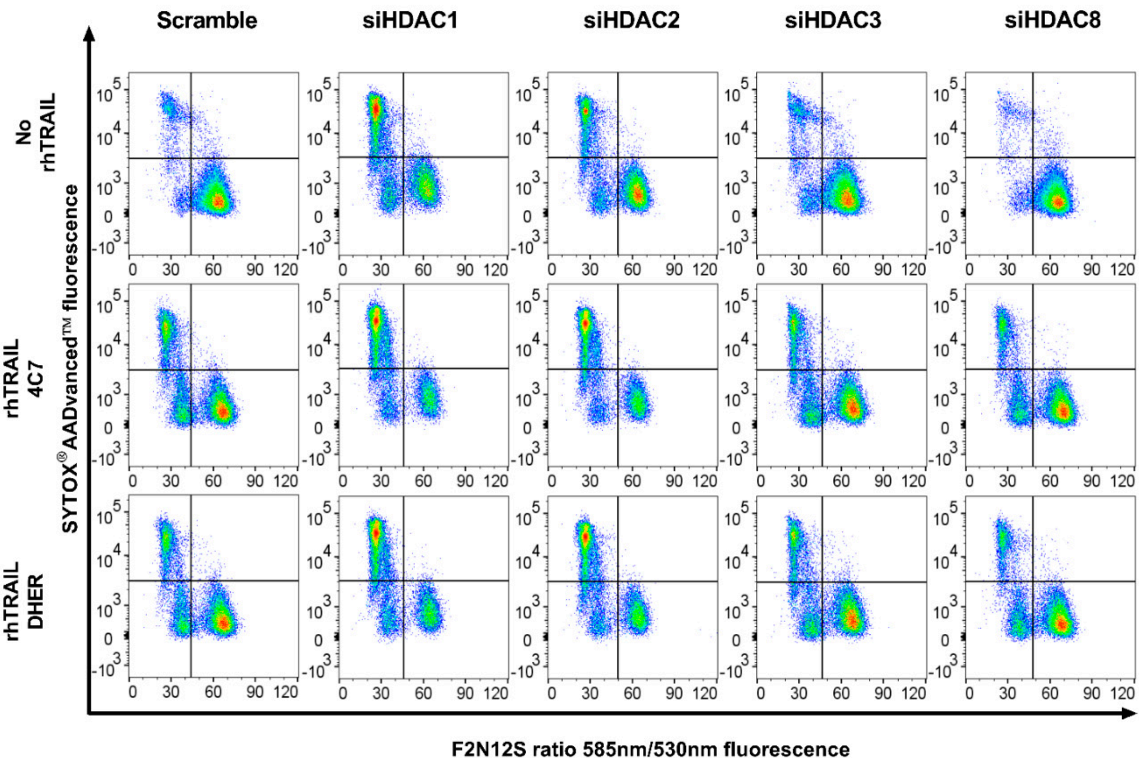

Figure S3. After 72 h transfection with siRNA, WiDr cells were treated with 25 ng/mL rhTRAIL 4C7 or DHER and apoptotic cells were detected using the Violet Ratiometric Membrane Asymmetry Probe.

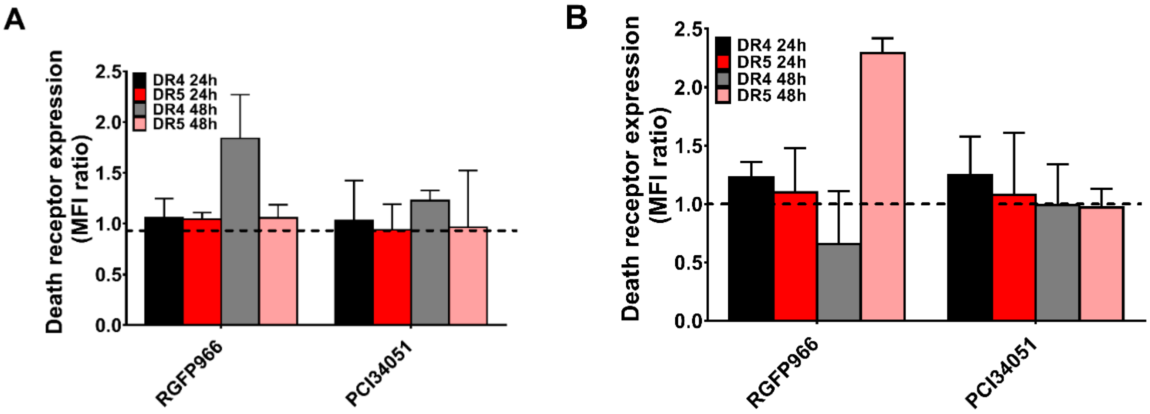

Figure S4. Alterations of death receptor expression after incubating with 10 μM RGFP966 or PCI34051 for 24 or 48 h on DLD-1 (**A**) or WiDr (**B**) cells.

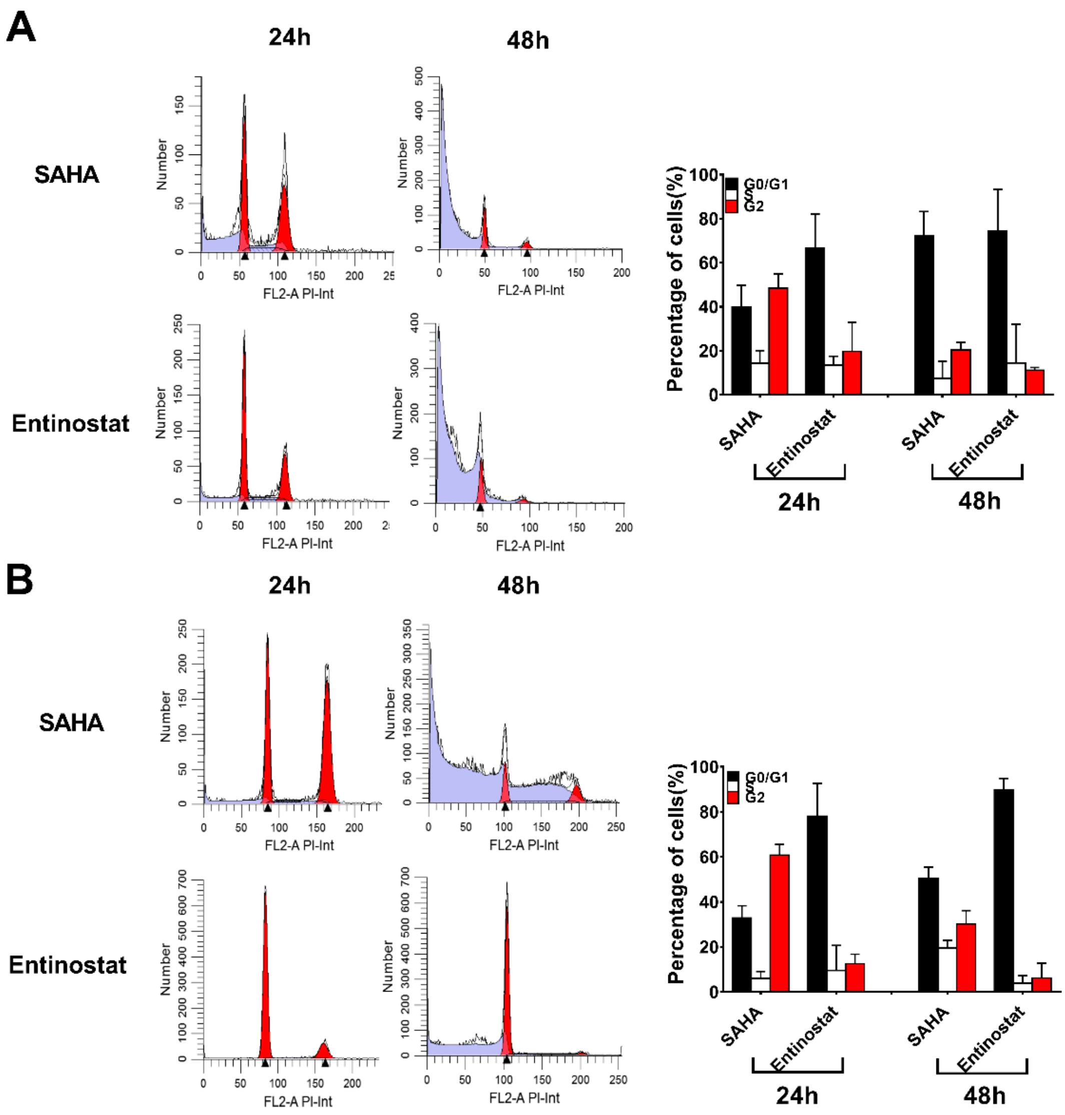

Figure S5. SAHA or Entinostat changes cell cycle after incubating for 24 h on DLD-1 (**A**) or WiDr (**B**) cells. Red peaks represent G0/G1 and G2 phase. Purple areas represent dead cells or debris. Statistical analysis on the right panel is according to the data on the left.

Table S1. List of primer sets used for qRT-PCR.

Name	Strand	Sequence
HDAC1	F	5′-AACAGGCCATCGAATACTGG-3′
	R	5′-GGAAATCTATCGCCCTCACA-3′
HDAC2	F	5′-AGACTGCAGTTGCCCTTGAT-3′
	R	5′-TGCGCAAATTTTCAAACAAA-3′
HDAC3	F	5′-TGGCTTCTGCTATGTCAACG-3′
	R	5′-CCCGGTCAGTGAGGTAGAAA-3′
HDAC8	F	5′- ATTCTCTACGTGGATTTGGATC-3′
	R	5′-ATGCCATCCTGAATGGGCACA-3′
α-tublin	F	5′-CTTCGTCTCCGCCATCAG-3′
	R	5′-CGTGTTCCAGGCAGTAGAGC-3′

© 2019 by the authors. Licensee MDPI, Basel, Switzerland. This article is an open access article distributed under the terms and conditions of the Creative Commons Attribution (CC BY) license (http://creativecommons.org/licenses/by/4.0/).
